# Force-of-infection of *Taenia solium* porcine cysticercosis: a modelling analysis to assess global incidence and prevalence trends

**DOI:** 10.1038/s41598-020-74007-x

**Published:** 2020-10-19

**Authors:** Matthew A. Dixon, Peter Winskill, Wendy E. Harrison, Charles Whittaker, Veronika Schmidt, Elsa Sarti, Saw Bawm, Michel M. Dione, Lian F. Thomas, Martin Walker, Maria-Gloria Basáñez

**Affiliations:** 1 Department of Infectious Disease Epidemiology and London Centre for Neglected Tropical Disease Research (LCNTDR), Faculty of Medicine, School of Public Health, Imperial College London, London, W2 1PG UK; 2 MRC Centre for Global Infectious Disease Analysis, Department of Infectious Disease Epidemiology, Faculty of Medicine, School of Public Health, Imperial College London, London, W2 1PG UK; 3 SCI Foundation, Edinburgh House, 170 Kennington Lane, London, SE11 5DP UK; 4 Department of Neurology, Center for Global Health, Technical University Munich (TUM), Munich, Germany; 5 Centre for Global Health, Institute of Health and Society, University of Oslo, Oslo, Norway; 6 Sanofi Pasteur Latin America, Av. Universidad N° 1738, Colonia Coyoacán, 04000 Mexico, D.F. Mexico; 7 University of Veterinary Science, Yezin, Nay Pyi Taw, 15013 Myanmar; 8 International Livestock Research Institute, 01 BP 1496, Ouagadougou, Burkina Faso; 9 International Livestock Research Institute (ILRI), Old Naivasha Road, PO Box 30709-00100, Nairobi, Kenya; 10 Institute for Infection and Global Health, University of Liverpool, 8 West Derby Street, Liverpool, L69 7BE UK; 11 Department of Pathobiology and Population Sciences and London Centre for Neglected Tropical Disease Research (LCNTDR), Royal Veterinary College, Hatfield, AL9 7TA UK

**Keywords:** Epidemiology, Mathematics and computing

## Abstract

The World Health Organization (WHO) called, in 2012, for a validated strategy towards *Taenia solium* taeniasis/cysticercosis control and elimination. Estimating pig force-of-infection (FoI, the average rate at which susceptible pigs become infected) across geographical settings will help understand local epidemiology and inform effective intervention design. Porcine cysticercosis (PCC) age-prevalence data (from 15 studies in Latin America, Africa and Asia) were identified through systematic review. Catalytic models were fitted to the data using Bayesian methods, incorporating uncertainty in diagnostic performance, to estimate rates of antibody seroconversion, viable metacestode acquisition, and seroreversion/infection loss. There was evidence of antibody seroreversion across 5 studies, and of infection loss in 6 studies measured by antigen or necropsy, indicating transient serological responses and natural resolution of infection. Concerted efforts should be made to collect robust data using improved diagnostics to better understand geographical heterogeneities in *T. solium* transmission to support post-2020 WHO targets.

## Introduction

The control and elimination of the zoonotic neglected tropical disease (zNTD) *Taenia solium* taeniasis/cysticercosis, presents a substantial public health challenge. The two-host *T. solium* lifecycle comprises definitive human hosts and intermediate pig hosts^1^. Humans become infected and develop taeniasis following ingestion of larval-stage metacestode cysts in under-cooked pork. Cysts evaginate in the intestine and develop into adult tapeworms containing immature, mature and gravid proglottid segments, with the gravid proglottids harbouring large numbers of eggs (3900–126,520)^2^. Release of gravid proglottids in human faeces exposes pigs to eggs either directly, by coprophagia, or indirectly, through environmental contamination by mechanical vectors or other dispersal mechanisms^3^. Ingestion of eggs by pigs enables mature oncospheres to migrate to internal organs and tissues, resulting in porcine cysticercosis (PCC). Humans can become accidental intermediate hosts, developing cysticercosis and specifically neurocysticercosis (NCC) through consumption of eggs by the faecal-oral route. NCC is one of the leading preventable causes of epilepsy and seizures in endemic settings^4^ across Meso and South America, sub-Saharan Africa, and Central and East Asia^5^. Surveys suggest that PCC prevalence ranges from 3 to 75%^6,7^ based on antibody detection (which measures exposure), 5–55%^8,9^ based on antigen detection (which measures active infection), and 0.1–29%^10,11^ (upper value from a survey in slaughter-age animals) based on direct observation of cysts through tongue inspection or necropsy/meat inspection. Human taeniasis prevalence, which is mainly measured by detection of antigen in stools, is generally low (on average less than 3%), with estimates ranging between 0 and 17% in endemic settings^12^, while human cysticercosis prevalence ranges from 4 to 7% based on antigen and 13–17% based on antibody detection^12^. In terms of disease burden related to NCC-associated epilepsy, a median of 2.8 million (95% uncertainty interval: 2.1–3.6 million) Disability-Adjusted Life Years (DALYs) was estimated globally in 2010 for T. solium^13^, although this figure is likely to be an underestimation as other neurological sequelae resulting from NCC are not considered^14^.An increasing, rapidly urbanizing and more affluent world population is driving demand for animal-source protein, and much of the ongoing and future demand will be met by meat from monogastric animals, particularly poultry and pigs^15^. Pigs have been a traditional component of household livelihoods across Latin America and South-East Asia and are playing an increasingly important role in the livelihoods of communities across endemic settings in sub-Saharan Africa (SSA)^16^. In these regions, smallholder and subsistence farmers often prefer pigs over other livestock because of their high fecundity and fast growth rates^17,18^. Pigs are also generally cheaper to purchase than other livestock with little or no additional feeding costs to the farmer (given pigs natural ability to scavenge)^19^. Pigs offer, therefore, an excellent investment, or source of emergency cash reserve. However, the free-roaming behaviour of pigs facilitates *T. solium* transmission where open defecation is common, often because of low or zero access to latrines. In SSA it is estimated that only 28% of the population have access to basic or safely managed sanitation facilities^20^. Even when latrine coverage is high, open defecation may still be practised^21^. Other practices such as direct feeding of human faeces to pigs may further increase exposure^22^

The identification of optimal and validated strategies for *T. solium* taeniasis/cysticercosis control and elimination, called for in the 2012 World Health Organization (WHO) roadmap on NTDs^23^, would be aided by a comprehensive global picture of *T. solium* transmission dynamics, aimed to understand whether and where geographical heterogeneity in transmission will require tailored setting-specific interventions. One crucial parameter is the Force-of-Infection (FoI), the average rate at which susceptible individuals become infected. The FoI can be estimated from the rate of seroconversion where a relationship exists between host age and seroprevalence^24^. An age-stratified seroprevalence survey in Ecuador was used to estimate the annual rate at which humans develop cysticercosis antibodies^25^. Although literature is available on age-seroprevalence patterns in pigs, FoI estimates for PCC are lacking.

Pig populations studied in Mozambique, Bolivia and Peru, have demonstrated an approximately linear relationship between cysticercosis seroprevalence and pig age^7,26–28^. This suggests that pigs may be exposed to *T. solium* eggs at a relatively constant rate in these settings. Pigs that have previously tested antibody-positive may, however, serorevert, with one study in Peru estimating that 20% of animals became antibody seronegative over a 4-month period^29^. These dynamics may result from waning maternal immunity, exposure without establishment of infection or through aborted mild infection^30^. In contrast to the variable antibody dynamics, it is generally assumed that the larval-stage metacestode infection in pigs is life-long, especially over the relatively short lifespans of pigs in endemic settings, as pigs are often slaughtered at an early age without chance to develop immunity and clear infection^31^. Non-viable, degenerated cysticerci have been found in necropsied pigs^32,33^, which may indicate that acquired protective immunity is possible from continued exposure to the parasite over a pig’s life-time. There is also evidence of immunity-mediated regulation in other Taeniidae species in their intermediate hosts. For example, sheep become resistant to *Taenia hydatigena* with age and maintain immunity by the ‘boosting effect’ of constant exposure^34^.

This study presents estimates of PCC FoI across a range of epidemiological and geographical settings, by conducting a systematic review of publicly (and solicited from authors) available data, and applying Bayesian methods to fit (simple and reversible) catalytic models to these data incorporating diagnostic uncertainty. The results improve understanding of geographical variation in transmission and will contribute to refining *T. solium* transmission models (e.g. cystiSim^35^, EPICYST^36^) by facilitating setting-specific model parameterisations to better reflect local epidemiological conditions. Ultimately, this will assist in the design of effective intervention strategies that are tailored to specific settings. The underlying epidemiological processes that shape age-(sero)prevalence relationships, including the potential role of acquired immunity and exposure heterogeneity are discussed.

## Results

### Study selection

**Figure 1:**
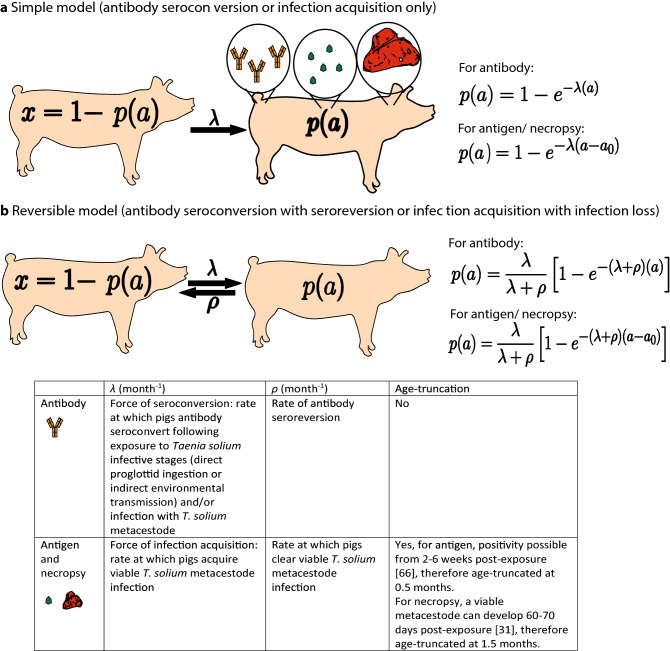
Simple and reversible catalytic model structure and equations of the models fitted to data on the age (*a*)-specific (sero)prevalence (*p*(*a*)), where *λ* is the force-of-infection (rate of seroconversion or infection acquisition) and *ρ* the rate of seroreversion or infection loss. The general mathematical form of the catalytic models fitted to the antibody (first equation in 1a and 1b), antigen and necropsy prevalence (second equation in 1a and 1b) datasets to estimate the prevalence (*p*) at pig age (*a*). Setting *a*_0_ = 0 yields the non-truncated model variant and setting *ρ* = 0 yields the non-reversible, simple catalytic model. The saturating (sero)prevalence is given by *λ*/(*λ* + *ρ*) which for the simple model is 100%, if the pigs lived sufficiently long. The accompanying tables provide information on the definitions of the catalytic model parameters depending on the diagnostic method used to detect positivity.

After title, abstract and full-text eligibility screening of 1809 studies initially identified in the search, a total of 15 studies were included in the analysis (Supplementary File Figure S1), originating from Latin America, Africa and Asia (Supplementary File Figure S2). Age-(sero)prevalence data were available directly from 12 published articles and data were obtained after contacting the authors of a further 3 articles. Eight studies used serology to detect PCC antibodies, four studies used serology to detect PCC antigen (Kungu et al.^37^ in Uganda stratified their data into urban and rural production systems using two different Ag-ELISA diagnostics), and the 3 remaining studies used necropsy to identify metacestode cysts. The sampling strategies in the various studies, where detailed information was available, indicated that pigs were either selected randomly (individual pigs or households), or all eligible pigs in a survey area were sampled in serological surveys (Supplementary File Table S1). For the 3 necropsy-based surveys, where information was available, sampling was based on pig age, which was either slaughter-age or pre-slaughter-age (Supplementary File Table S1). Models (see “Methods” for schematic representation of catalytic model structure, Fig. 1) were fitted to observed (sero)prevalence data using a Bayesian framework, integrating prior (published) information on the sensitivity and specificity of each diagnostic test. Where the same diagnostic was used across multiple surveys, the diagnostic parameters were jointly fitted across datasets (estimating a single posterior distribution for sensitivity and specificity across datasets), while FoI parameters (*λ* and *ρ*) were estimated for each dataset.

The deviance information criterion (DIC) was used to compare model fits for individually- or jointly-fitted datasets. For the purpose of this analysis, we define studies with overall *observed* pig cysticercosis (sero)prevalence as hypoendemic (0–9.99%), mesoendemic (10–24.99%) or hyperendemic (≥ 25%) transmission settings. Supplementary File Table S1 details included studies, sampling strategies, diagnostics used and construction of priors.

A more intuitive approach to understanding the FoI parameter *λ* is to consider its reciprocal which here corresponds to the average time until pigs become antibody seropositive or infected (measured by antigen or necropsy data). Equally, the reciprocal of parameter *ρ* relates to the average duration that pigs remain antibody positive or infected. These values, obtained from selected models (based on the DIC), are compared across settings (by all-age (sero)prevalence of each dataset and by country).

### Antibody seroprevalence

**Figure 2:**
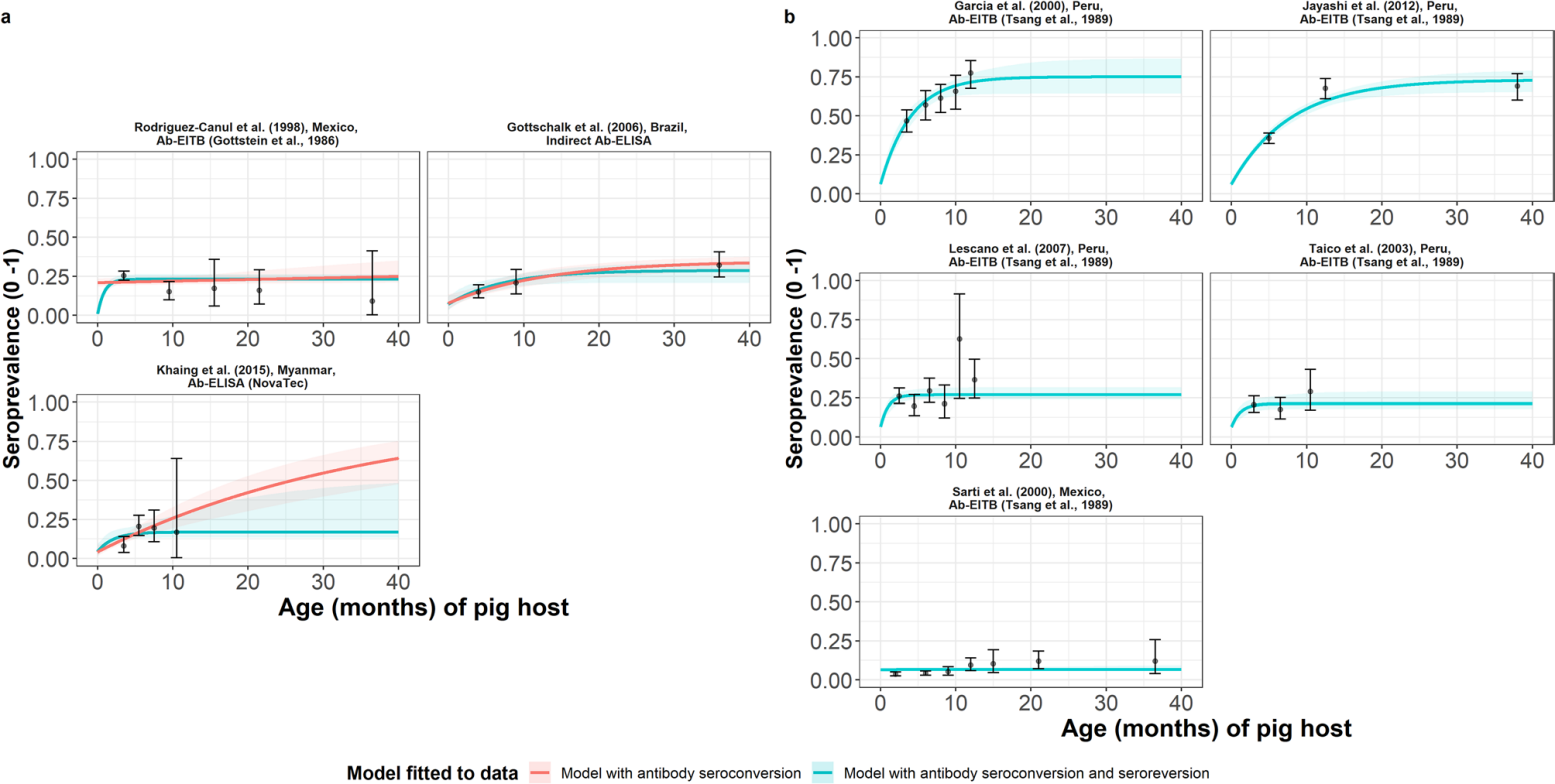
The relationship between antibody seroprevalence and pig age (in months) for each dataset. Antibody seroconversion (simple) or seroconversion with seroreversion (reversible) catalytic models for (**a**) individually-fitted datasets and (**b**) jointly-fitted datasets (single diagnostic sensitivity and specificity values estimated; dataset-specific *λ* and *ρ* estimates obtained), including 95% confidence intervals associated with observed antibody seroprevalence point estimates. Bayesian Markov chain Monte Carlo methods were used to fit the models to data, with the parameter posterior distributions used to construct predicted (all age) seroprevalence curves and associated 95% Bayesian credible intervals (BCIs). Best-fitting model selected by deviance information criterion (DIC); both models presented if difference between DIC < 2 (both models have similar support based on the data); a difference > 10 units indicates that the models are significantly different and therefore only superior fitting model (lowest DIC) is presented). The non-zero predicted seroprevalence at age 0 is due to less than 100% specificity for all tests. The 95% confidence intervals (95% CI) for age-seroprevalence data-points are calculated by the Clopper-Pearson exact method.

Table 1 presents the results of the best model fits, either including (reversible model) or excluding (simple model) seroreversion. For the jointly-fitted datasets (all using the antibody lentil lectin-purified glycoprotein enzyme-linked immunoelectrotransfer blot (LLGP-EITB) assay^38,39^), the antibody seroconversion with seroreversion model provided a more adequate fit according to the DIC (Table 1, Fig. 2a). These studies were found across hypo- (all-age seroprevalence of 5.3% in Mexico^40^), meso- (all-age seroprevalence of 20.7% in Peru^41^), and hyperendemic settings (all-age seroprevalence of 26.2–58.8% in Peru^7,42,43^). For the individually-fitted datasets, antibody seroconversion-only models were preferred (Table 1, Fig. 2b), with all studies found in the mesoendemic range (all-age seroprevalence of 15.9–23% for Myanmar^44^, Brazil^45^ and Mexico^46^). In the case of Sarti et al.^40^ and Rodriguez et al.^46^ data, both collected in Mexico, the estimated average time until becoming antibody seropositive (1/λ) exceeded the maximum expected natural lifespan of pigs (180 months for *Sus scrofa*^47^). This indicates that it was not possible to distinguish true positives from false positives in these low endemic settings (i.e. the data are consistent with a negligibly low FoI) and the FoI estimates are, therefore, not presented. Supplementary File Table S2 presents all model fits and DIC scores for each dataset.

**Table 1:** Seroprevalence and parameter estimates for the best-fit catalytic models fitted to each observed antibody age-seroprevalence dataset (ordered by decreasing all-age seroprevalence).

Dataset, country	All-age observed sero-prevalence (%)	Best-fit catalytic model	Diagnostic sensitivity (95% BCI)	Diagnostic specificity (95% BCI)	*λ* = seroconversion rate, month^−1^ (95% BCI)	*ρ* = seroreversion rate, month^−1^ (95% BCI)
**Jointly-fitted datasets**^**a**^						
Garcia et al. 2003^7^ Peru	58.8	Reversible^b^	0.889 (0.749–0.991)	0.936 (0.925–0.946)	0.207 (0.147–0.318)	0.042 (0.004–0.124)
Jayashi et al.^42^ Peru	45.2				0.104 (0.085–0.133)	0.024 (0.004–0.049)
Lescano et al. ^43^ Peru	26.2				0.247 (0.116–0.387)	0.746 (0.280–0.986)
Taico et al.^41^ Peru	20.7				0.152 (0.063–0.269)	0.692 (0.209–0.984)
Sarti et al.^40^ Mexico	5.3				0.001 (0.00006–0.007)	0.63 (0.022–0.980)
**Individually-fitted datasets**						
Rodriguez-Canul et al.^46^ Mexico	23.02	Simple^c^	0.940 (0.806–0.990)	0.790 (0.765–0.82)	0.001 (0.0001–0.006)	NA
Gottschalk et al.^45^ Brazil	20.5	Simple^c^	0.349 (0.297–0.403)	0.921 (0.868–0.963)	0.078 (0.035–0.146)	NA
Khaing et al.^44^ Myanmar	15.9	Simple^c^	0.940 (0.888–0.973)	0.958 (0.915–0.985)	0.028 (0.015–0.040)	NA

Parameter median posterior estimates are presented with 95% Bayesian credible intervals (95% BCI). Supplementary File Table S1 provides full (location, diagnostics) details of studies.

*NA* not applicable.

^a^Diagnostic sensitivity and specificity for the antibody lentil lectin-purified glycoprotein enzyme-linked immunoelectrotransfer blot (Ab LLGP-EITB) assay^38,39^ were jointly fitted across datasets.

^b^Best-fitting model determined by DIC (jointly-fitted dataset).

^c^Best-fitting model determined by DIC (individually-fitted dataset).

### Antigen seroprevalence

**Figure 3:**
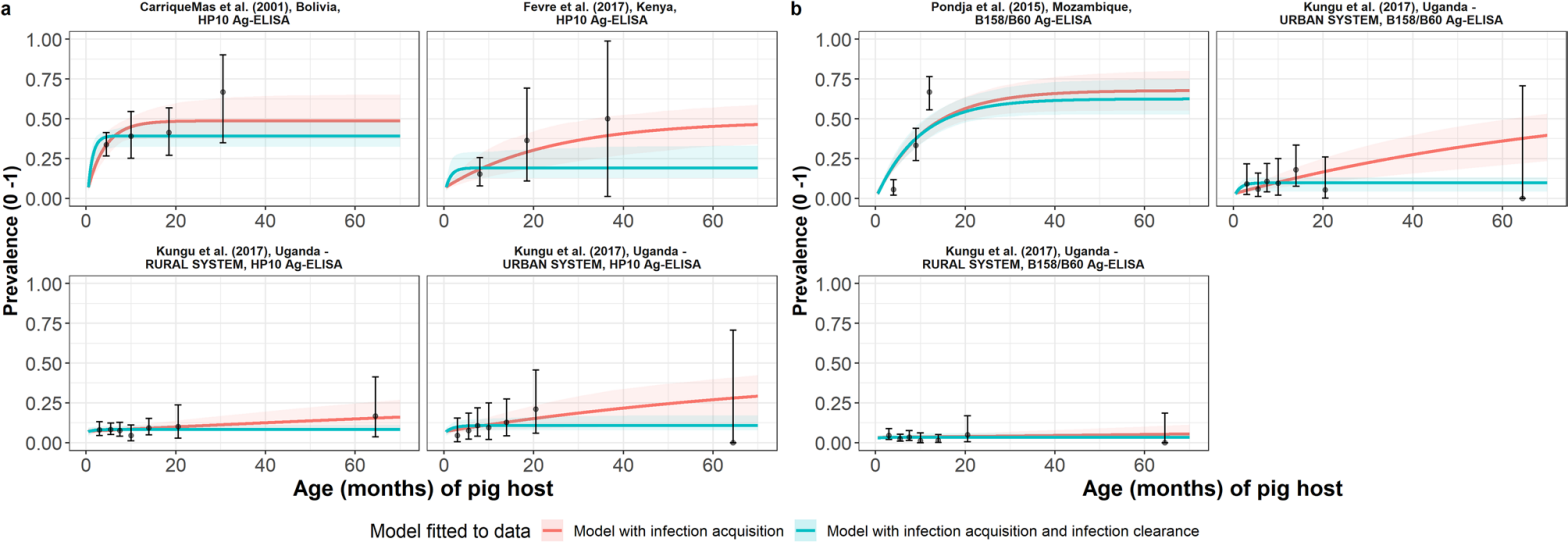
The relationship between antigen seroprevalence and pig age (in months) for (**a**) Carrique-Mas et al.^27^ in Bolivia; Pondja et al.^51^ in Mozambique; Fèvre et al.^49^ in Kenya; and (**b**) Kungu et al.^37^ in urban- and rural-production systems in Uganda. Viable *Taenia solium* metacestode infection acquisition models with (reversible) or without (simple) infection loss jointly-fitted to antigen seroprevalence datasets (single diagnostic sensitivity and specificity values estimated; dataset-specific *λ* and *ρ* estimates obtained) for (**a**) HP10 Ag-ELISA and (**b**) B158/B60 Ag-ELISA or commercial B158/B60 Ag-ELISA (apDia, Turnhout, Belgium), including 95% confidence intervals associated with observed antigen seroprevalence point estimates. Bayesian Markov chain Monte Carlo methods were used to fit the models to data, with the parameter posterior distributions used to construct predicted prevalence curves and associated 95% Bayesian credible intervals (BCI). Best-fitting model selected by deviance information criterion (DIC); both models presented if difference between DIC < 2 (both models have similar support based on the data); a difference > 10 units indicates that the models are significantly different and therefore only superior fitting model (lowest DIC) is presented). In Kungu et al.^37^ (Uganda) model-predicted prevalence is presented based on the urban- and rural-stratified data. The non-zero predicted seroprevalence at age 0 is due to less than 100% specificity for all tests. The 95% confidence intervals (95% CI) for age-seroprevalence data-points are calculated by the Clopper-Pearson exact method.

Infection acquisition with viable *T. solium* metacestode infection (simple model) provided a more adequate fit (Table 2, Fig. 3a) for the jointly-fitted datasets (based on using the HP10 Antigen- enzyme-linked immunosorbent assay, Ag-ELISA^48^), with studies found across hypoendemic (all-age seroprevalences of 8.1–9.8% in a Ugandan rural and urban production system^37^), mesoendemic (all-age seroprevalence of 18.8% in Kenya^49^) and hyperendemic settings (all-age seroprevalence of 37.4% in Bolivia^27^). Jointly-fitted datasets based on the B158/B60 Ag-ELISA^50^ or commercial B158/B60 Ag-ELISA (apDia, Turnhout, Belgium) indicate that the infection acquisition and loss of viable *T. solium* metacestode infection (reversible) model is preferred (Table 2, Fig. 3b) for hypoendemic (all-age seroprevalence of 2.9–9.8% in a Ugandan rural and urban production system^37^), and hyperendemic settings (all-age seroprevalence of 32.6% in Mozambique^51^). The average time until becoming infected (1/*λ*) exceeded the maximum expected natural lifespan of pigs for the Ugandan rural production system that used the HP10 Ag-ELISA^37^ and B158/B60 Ag-ELISA^37^ assays. Supplementary File Table S3 presents all model fits and DIC scores for each dataset.

**Table 2:** Seroprevalence and parameter estimates for the best-fit catalytic models fitted to each observed antigen age-seroprevalence dataset (ordered by decreasing all-age seroprevalence).

Dataset, country	All-age observed sero-prevalence (%)	Best-fit catalytic model	Diagnostic sensitivity (95% BCI)	Diagnostic specificity (95% BCI)	*λ* = rate of infection acquisition, month^−1^ (95% BCI)	*ρ* = rate of infection loss, month^−1^ (95% BCI)
**Jointly-fitted datasets**^**a**^						
Carrique-Mas et al.^27^ Bolivia	37.4	Simple^c^	0.488 (0.376–0.650)	0.927 (0.907–0.949)	0.254 (0.109–0.836)	NA
Fèvre et al.^49^ Kenya	18.8				0.042 (0.016–0.105)	NA
Kungu et al.^37^ (urban) Uganda	*HP10*: 9.8				0.011 (0.0015–0.029)	NA
Kungu et al.^37^ (rural) Uganda	*HP10:* 8.11				0.003 (0.0004–0.011)	NA
**Jointly-fitted datasets**^**b**^						
Pondja et al.^51^ Mozambique	32.6	Reversible^c^	0.685 (0.552–0.815)	0.970 (0.956–0.981)	0.093 (0.067–0.143)	0.009 (0.0005–0.042)
Kungu et al.^37^ (urban) Uganda	*B158/B60*: 9.8				0.079 (0.020–0.186)	0.677 (0.112–0.984)
Kungu et al.^37^ (rural) Uganda	*B158/B60:* 2.85				0.005 (0.0003–0.024)	0.733 (0.122–0.988)

Parameter median posterior estimates are presented with 95% Bayesian credible intervals (95% BCI). Supplementary File Table S1 provides full (location, diagnostics) details of studies.

*NA* Not applicable.

^a^Diagnostic sensitivity and specificity for the HP10 antigen- enzyme-linked immunosorbent assay (Ag-ELISA) test^48^ was jointly fitted across datasets.

^b^Diagnostic sensitivity and specificity for the B158/B60 Ag-ELISA^50^ or commercial B158/B60 Ag-ELISA (apDia, Turnhout, Belgium) was jointly fitted across datasets.

^c^Best fitting model determined by DIC (jointly-fitted dataset).

### Necropsy

**Figure 4:**
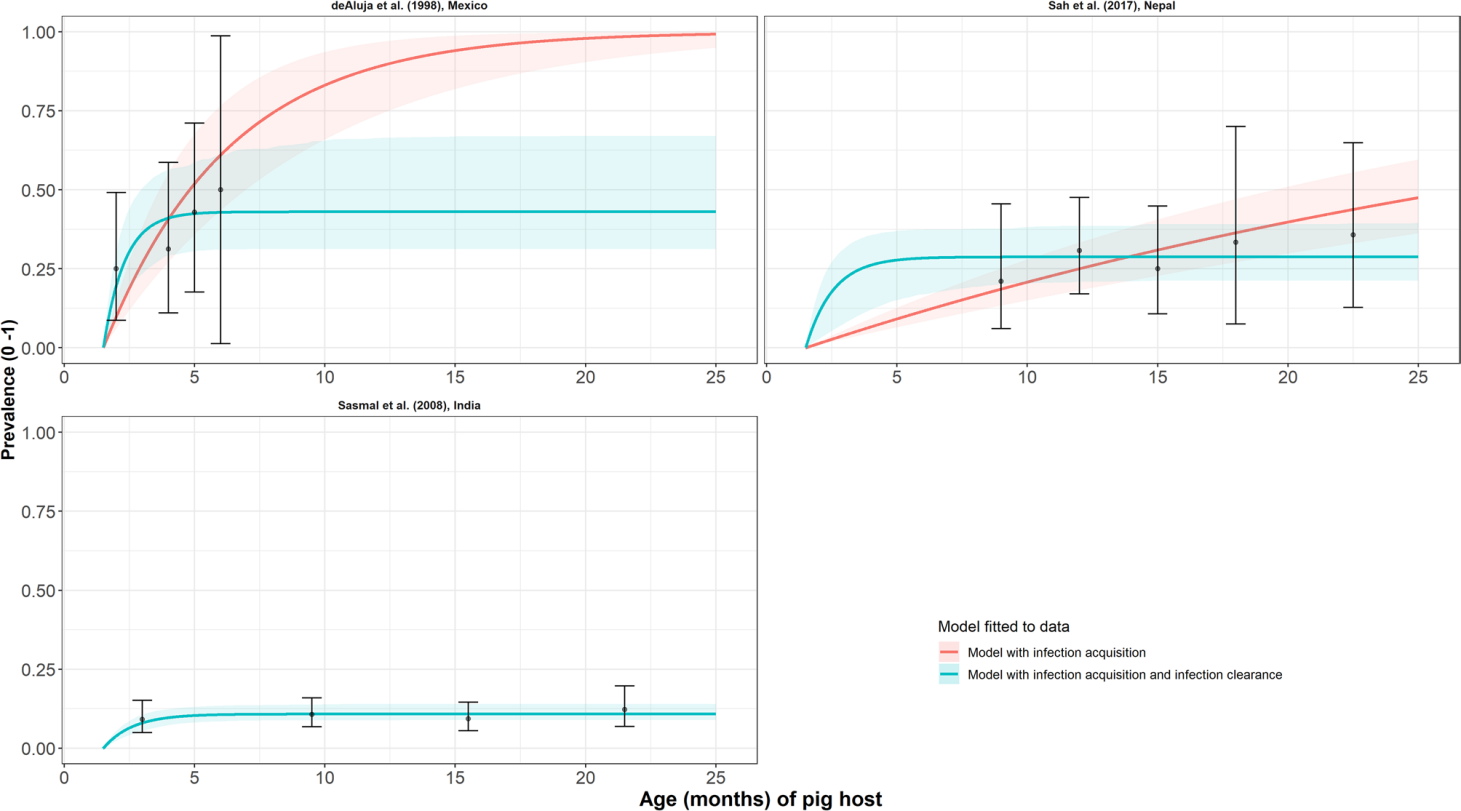
The relationship between necropsy prevalence and pig age (months) for each dataset. Viable *Taenia solium* metacestode infection acquisition models with (reversible) or without (simple) infection loss fitted to each necropsy age-prevalence dataset, including 95% confidence intervals associated with observed prevalence point estimates. Bayesian Markov chain Monte Carlo methods were used to fit the models to data, with the parameter posterior distributions used to construct predicted prevalence curves and associated 95% Bayesian credible intervals (BCI). Best-fitting model selected by deviance information criterion (DIC); both models presented if difference between DIC < 2 (both models have similar support based on the data); a difference > 10 units indicates that the models are significantly different and therefore only superior fitting model (lowest DIC) is presented). The 95% confidence intervals (95% CI) for age-prevalence data-points are calculated by the Clopper-Pearson exact method.

Across meso- and hyperendemic transmission settings (all-age prevalence settings of 10.3–32.7% in India^52^, Nepal^32^ and Mexico^53^), infection loss following viable *T. solium* metacestode infection (reversible model) was identified as the best-fitting model (Table 3, Fig. 4). There was very similar support (less than 1 DIC unit difference) for both (simple and reversible) models in a hyperendemic setting in Nepal^32^ (all-age prevalence of 28.4%). Supplementary File Table S4 presents all model fits and DIC scores for each dataset.

**Table 3:** Prevalence and parameter estimates for the best-fit catalytic models fitted to each observed necropsy age-prevalence dataset (ordered by decreasing all-age prevalence).

Dataset, country	All-age observed prevalence (%)	Best-fit catalytic model	*λ* = rate of infection acquisition, month^−1^ (95% BCI)	*ρ* = rate of infection loss, month^−1^ (95% BCI)
de Aluja et al.^53^ Mexico	32.7	Reversible^a^	0.529 (0.245–0.896)	0.700 (0.163–0.986)
Sah et al.^32^ Nepal	28.4	Reversible^a^	0.276 (0.058–0.515)	0.684 (0.133–0.980)
Sasmal et al.^52^ India	10.3	Reversible^a^	0.097 (0.052–0.137)	0.801 (0.418–0.986)

Parameter estimates are summarized by the median and 95% Bayesian credible interval (95% BCI) of the posterior distribution. Supplementary File Table S1 provides full (location) details of the studies. Diagnostic sensitivity and specificity parameter estimates are not shown because fitting to uncertainty in necropsy diagnostic characteristics was not required (sensitivity and specificity were assumed to be 100%).

^a^Best fitting model determined by DIC (individually-fitted dataset).

### Force-of-infection across settings

**Figure 5:**
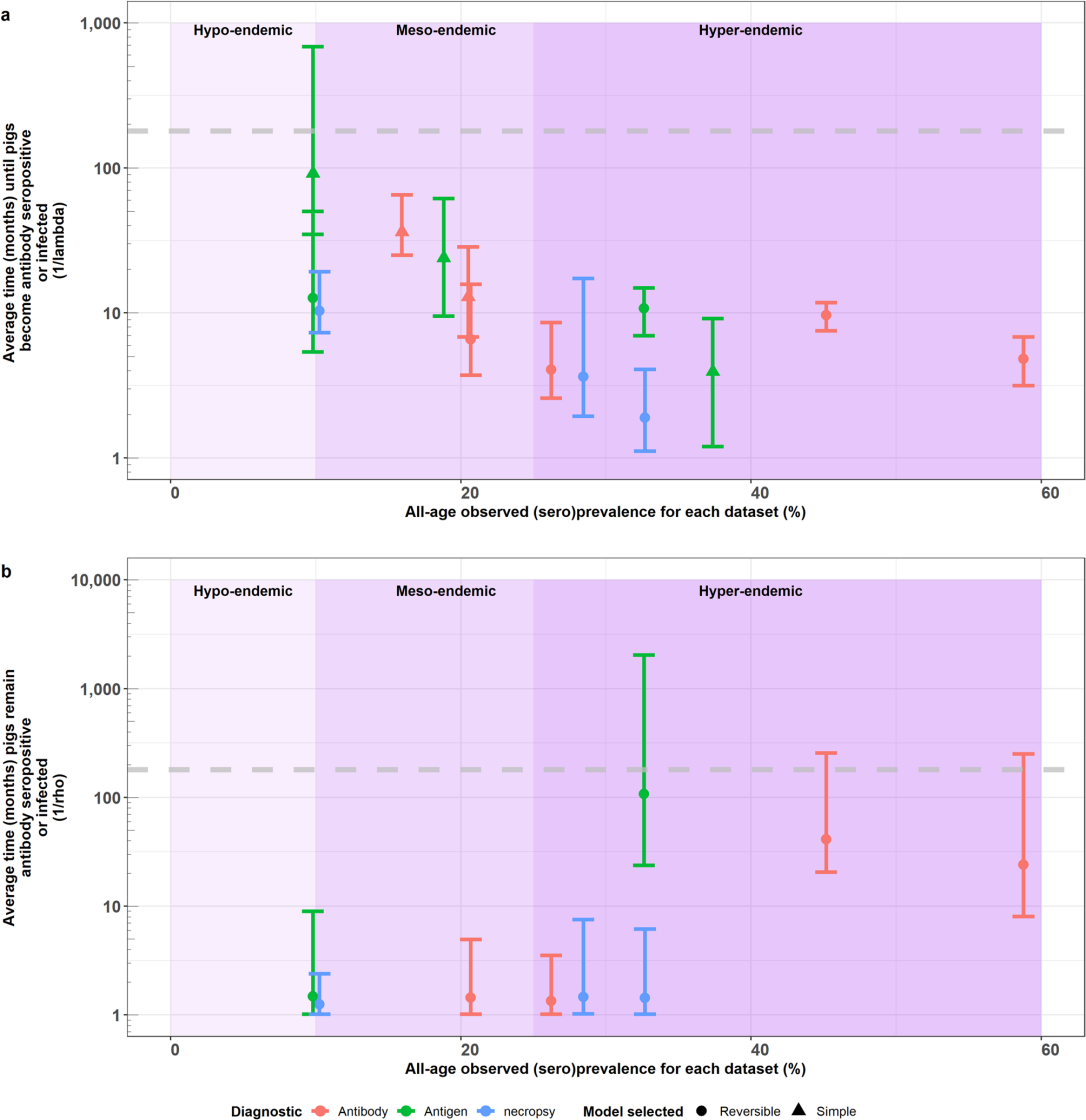
Average time (months) until pigs become antibody seropositive/infected (1/*λ*), or remain antibody seropositive or infected (1/*ρ*) vs. overall (all age) prevalence (percent). The relationship between (**a**) the average time until pigs become antibody seropositive or infected (1/λ) and overall (all-age) prevalence, and (**b**) the average time pigs remain antibody seropositive or infected (1/ρ) and overall (all-age) prevalence. The plot is stratified by proposed endemicity levels defined as hypoendemic (0–9.99% all-age (sero)prevalence), mesoendemic (10–24.99% all-age (sero)prevalence) and hyperendemic (≥ 25% all-age (sero)prevalence). Only λ median estimates are presented where 1/λ (average duration of susceptibility in months) is less than life expectancy of pigs; horizontal (grey) dashed line represents maximum life expectancy of pigs: 15 years × 12 months = 180 months^47^. The y-axis is in log scale for both panels.

Figure 5a illustrates an overall decline in the average time until pigs become antibody seropositive or infected with increasing all-age (sero)prevalence, with average estimates of < 10.7 months in hyperendemic settings, < 36.2 months in mesoendemic settings, and < 91.8 months in hypoendemic settings. In mesoendemic settings, one estimate (from Mexico^46^), and in hypoendemic settings, 3 estimates (from Uganda^37^ and Mexico^40^) exceeded the expected natural lifespan of pigs, 180 months (not shown in Fig. 5a) . There is no clear trend between an increasing all-age (sero)prevalence and the average duration that pigs remain antibody positive or infected (Fig. 5b). Higher average estimates (> 30.8 months) were found in the upper end of hyperendemic settings (> 33% all-age (sero)prevalence), but six, considerably smaller estimates (1.2–1.5 months) were found across hypo-, meso-, and hyperendemic settings (9.8–32.7% all-age (sero)prevalence). The reciprocals of *λ* and *ρ* estimates are presented in Supplementary File Tables S2–S4.

Across countries, there was significant variation in the duration until pigs become antibody positive or infected (Supplementary Figure S3). Within-country estimates were similar (likely due to using the same assays), as in Peru^7,41–43^ from reversible models (Supplementary Figure S3a). Two estimates in Uganda from the urban production systems, showed large variation between the reversible model (B158/B60 Ag-ELISA^37^) and the simple, no infection loss, model (HP10 Ag-ELISA^37^). (Note that the rural production system provided estimates exceeding pig life expectancy and are not presented.) For the average duration of being seropositive or infected (Supplementary Figure S3b), there was consistency across most countries with very low estimates, but higher estimates were identified in three settings for Mozambique^51^ and Peru^7,42^.

## Discussion

This paper presents the first estimates of the FoI of *T. solium* PCC across a range of epidemiological settings. Catalytic models to estimate FoI from age (sero)prevalence profiles have been used in other NTDs (e.g. Chagas disease, trachoma) to investigate spatial heterogeneity and temporal incidence trends (secular or elicited by interventions)^55–57^. The FoI estimates in this study show variation among settings (between and within countries), reinforcing the importance of understanding local epidemiological dynamics for the parameterisation of mathematical transmission models^58^ and the implementation of tailored, setting-specific intervention strategies^59^. A preliminary characterisation of different endemicity settings is also postulated, identifying that PCC FoI estimate ranges based on observed data are 0.0009–0.077 month^−1^ for hypoendemic settings (0–9.99% all-age (sero)prevalence); 0.002–0.276 month^−1^ for mesoendemic settings (10–24.99% all-age (sero)prevalence), and 0.097–0.529 month^−1^ for hyperendemic settings (≥ 25% all-age (sero)prevalence). More work is required to build a consensus towards characterising differing endemic settings for *T. solium*. In other NTDs, these are linked to morbidity for onchocerciasis (e.g. prevalence of blindness for onchocerciasis)^60^, and of trachomatous inflammation–follicular and trichiasis for trachoma prevalence^61^). This is an important next step for the *T. solium* research and implementation community given that the new WHO post-2020 NTD goals are stated in terms of achieving intensified control in “hyperendemic” settings^62^.

For PCC, an age-independent FoI was assumed given the observation of an approximately linear relationship between seroprevalence and (typically young) pig age^7,26–28^, although it has been noted that older pigs may have a higher chance or frequency of accessing human faeces due to hierarchal population structures^63,64^. The age-prevalence profiles from antibody-, antigen-, and necropsy-based datasets collated for this study generally suggested that a constant FoI was a reasonable, simplifying assumption. The range of surveys based on different diagnostics represents measurement of different epidemiological processes, with antibody positivity indicating exposure, given the difficulty in relating antibody responses to active cysticercosis infection in pigs. Validation studies using the LLGP-EITB assay^38,39^ to detect antibodies against larval antigen have indicated that identification of multiple bands are required for “ruling in” the presence of infection^65^. However, most historical surveys using the LLGP-EITB assay use the threshold of one diagnostic band to measure positivity, including the surveys incorporated in this analysis.

Another key element of this study was to investigate whether there was a strong signal for seroreversion (in the case of antibody-based surveys), and for infection loss (antigen- or necropsy-based surveys) across surveys. While antigen-based FoI estimates are interpreted as representing infection acquisition and infection loss in this analysis, it should be noted that antigen positivity can result from the presence of excretory/secretory (ES) products from immature metacestodes (developing from 2–6 weeks post-infection^66^) which may not establish as a fully viable infection, thus potentially representing a transient response to exposure rather than infection. The relative magnitude of seroreversion compared to seroconversion can provide insight into the stability of antibody responses, which in turn can begin to illuminate the underlying biological mechanisms governing parasite establishment and immunity. We did not test an age-dependent seroreversion assumption in our models, as processes such as immunosenescence in older individuals (suggested for human cysticercosis)^25,67^ would be minimal at a population level because pigs are often slaughtered at < 1 year of age. Moreover, a more complex age-dependent infection loss model, which would capture increased resistance in older animals resulting from repeat exposures (as explored for other cestodes such as *Echinococcus granulosus*)^34^ would likely be challenging to fit to limited datasets. Poudel et al.^11^, using necropsy data from Nepal suggest that pigs older than 1 year of age are relatively resistant to infection; however, to test this hypothesis with the datasets presented here would be difficult given the paucity of data and small sample sizes for older animals. Disentangling exposure heterogeneity from immunity is also challenging, as older pigs, particularly sows may be less mobile and therefore less exposed compared to younger animals (UC Braae, personal communication).

In the antibody serology-based datasets analysed in this paper, antibody seroreversion (reversible models) was a component of the best-fitting model for the jointly-fitted LLGP-EITB antibody datasets^7,41–43^ in Peru and Mexico^40^. In hyperendemic settings^7,42,43^, parameter *ρ* had well-defined posterior distributions, providing an average duration of pigs remaining antibody positive ranging from 1.4–41.1 months (Fig. 5b), suggesting that some antibody seroreversion may be occurring. In these settings therefore, intense exposure may manifest as transient antibody (or antigen for antigen-serology) responses, underpinned by the presence of partial establishment of infection as proposed by Nguekam et al.^66^. For the remaining hypo- or mesoendemic settings, characterised by flatter, less well-defined age-prevalence profiles, and when the model with antibody seroreversion was preferred, very large *ρ* estimates were obtained, with poorly-defined posterior distributions pushing against the upper limit of 1 month^–1^ (pigs are not expected to be seropositive for less than 1 month i.e. 1/*ρ*). Substantial uncertainty was associated with the *ρ* posterior distributions especially in these hypo- and mesoendemic settings , indicating that there is little information in the datasets to clearly determine the *ρ* parameter. Robust sampling at the lower pig age-range to characterise a distinct age-(sero)prevalence profile would be essential for informing model fits. Sampling across age ranges was generally sparse (and seroprevalence uncertain due to small sample sizes) in the obtained studies, making it difficult to clearly differentiate between the simple and reversible models and to identify seroreversion rates in some datasets.

For certain transmission (hypoendemic) settings characterised by flat age-(sero)prevalence profiles, especially where the FoI/seroconversion is low (Sarti et al.^40^ in Mexico; Kungu et al.^37^ in rural Ugandan production systems), it is not possible to be certain that pigs were exposed at all due to the likelihood of substantial numbers of false positives (not being possible to distinguish the infection model/process with only false-positives driving the model fit to observable data). Current serology-based diagnostics suffer from reduced specificity due to the presence of cross-reactions to *T. hydatigena* with the Ag-ELISA tests^68,69^ and specifically to the GP50 band in the antibody LLGP-EITB assay^70,71^. The modelling approach in this paper is based on fitting directly to observed data, rather than fitting to adjusted data, to allow incorporation of uncertainty associated with the sensitivity and specificity of the respective diagnostics into the relationship between the true prevalence (a function of the catalytic models) and the observed data. FoI and (sero)reversion estimates therefore reflect additional uncertainty generated by the limitation in the diagnostics. However, it is clear in hypoendemic settings, and even in settings with higher all-age seroprevalence, that suboptimal performance, especially surrounding assay specificity is a major barrier to understanding FoI dynamics.

Sensitivity of serological diagnostics can also be influenced by intensity of infection, as demonstrated for the B158/B60 Ag-ELISA in Zambia^72^. A mathematical relationship can be shown to exist between the prevalence and intensity of infection by assuming an overdispersed (negative binomial) distribution of *T. solium* larvae in pig populations. To potentially characterise this relationship, and derive an expression relating sensitivity to prevalence, matched data on infection prevalence and intensity across a wide range of settings would be needed with which to estimate the overdispersion parameter. Current estimates of overdispersion in the parasite distribution among pig hosts (*k* = 0.23–0.37) come from a single and small-scale study in Mexico^73^*.* In addition, aggregated seroprevalence estimates obtained from systems where pigs are kept in different ways, such as in Uganda^37^, where pigs are kept either as “tethered” or “free-range”, could mask specific age-seroprevalence profiles. Clustering of PCC infection has also been documented in SSA^74^ and Latin American settings^75^, further highlighting that population prevalence surveys may miss these dynamics in the absence of additional spatial analysis.

Inclusion of prevalence data obtained from necropsy studies, considered the most accurate diagnostic for PCC, is an important aspect of our study. All three necropsy-based studies suggested that the best-fit model included infection loss, particularly (according to the DIC) the data from India^52^. For antigen-based data, best fit models including infection loss were identified for 3 datasets (jointly-fitted B158/B60 Ag-ELISA datasets in Mozambique^51^ and rural and urban production system stratified data for Uganda^37^). Our model assumed a constant rate of infection loss, so we cannot say whether age-dependent resistance potentially acted as a driving mechanism following repeat exposures. As previously described, insufficient resolution in the datasets, particularly necropsy-based datasets, prevents testing of this hypothesis. While necropsy is considered the most accurate diagnostic method, it is still imperfect, particularly because small cysts and light infections can be missed^76^. It is also difficult to determine the completeness of necropsy/dissection protocols in older studies. Additionally, sampling limitations due to cost and logistical barriers mean that age ranges of necropsied pigs are not necessarily representative, as younger pigs may be missed when sampling pigs close to slaughter age or weight^32,52^, or conversely, when it is difficult to purchase older pigs from farmers as they near slaughter age^53^. Limitations with the current necropsy-based datasets in particular highlight the need to collect age-prevalence and intensity data which are more representative across age groups and include larger sample sizes, such as the datasets obtained for other cestode infections^77^. This will facilitate fitting models of greater complexity, such as those including acquired immunity^77^. Not only will be a quantification of immunity important when modelling the impact of interventions, but also omission of immunity, if present, may lead to an underestimation of the FoI^34^.

The results presented here synthesize available literature and data to estimate the FoI of PCC across a variety of geographical and epidemiological settings globally, alongside preliminary construction of characteristics that could be used to define different endemicity levels for *T. solium*. There is support for transient antibody serological responses, and evidence for loss of viable cyst infection; however, limitations associated with the available data and sub-optimal diagnostics pose significant constraints to PCC FoI estimation and model testing. While the age-(sero)prevalence profiles are not suggestive of specific age-infection dynamics, transmission dynamics models still require age-structured pig populations to investigate realistic field-interventions (e.g. TSOL18 vaccination scheduling and minimal intervention strategies^31^). Modelling the potential impact of such interventions, in a variety of geographical and epidemiological settings, is a critical step to supporting the development and monitoring of post-2020 WHO NTD goals for *T. solium*, in particular the prospective goals of achieving “intensified control in hyperendemic areas”. Therefore, this analysis does not only suggest that different epidemiological settings will likely require tailored interventions, but it may be possible to identify different *T. solium* endemicity zones based on understanding the FoI trends (and hence characterising “hyperendemic areas”). Complementing this work with FoI estimation in human taeniasis and cysticercosis will also facilitate parameterisation of pig-to-human and human-to-pig transmission coefficients for mathematical models of *T. solium* taeniasis/cysticercosis^35,36^.

## Methods

### Identifying relevant literature, data sources and data extraction

Published articles with PCC age-(sero)prevalence *or* age-infection data suitable for constructing age-stratified profiles were identified through a systematic search conducted following the PRISMA guidelines^78^, adapted from a previous systematic review which gathered human *T. solium* cysticercosis and taeniasis serological data^12^. Dates for the literature search spanned between 31/12/1988 and 30/04/2018. PRISMA flowchart and search results are in Supplementary Figure S1. Identified studies are summarized in Supplementary Table S1; the geographical distribution of the data is presented in Supplementary Figure S2. The observed age-(sero)prevalence profiles were extracted from the articles identified from the systematic literature search or calculated from individual-level pig infection datasets after successful contact with study authors.

### Force-of-infection modelling

The FoI describes the average (per capita) rate at which susceptible individuals become infected. Multiplying this quantity by the total number of susceptible individuals in a population gives the incidence rate. The catalytic family of models, originally described by Muench^79^, considers the rate of conversion from susceptible to infected, and has been used to estimate the FoI by quantifying the rate of change in the proportion of infected individuals with age, using age-specific seroprevalence or infection data^80^. An important assumption for the simplest model is that this rate remains constant with respect to age (although age-varying FoI can also be implemented)^81,82^.

Catalytic models (Fig. 1) were used to estimate either the rate of antibody seroconversion (*λ*) and seroreversion (*ρ*) from the antibody age-seroprevalence data, or the rate of acquisition (*λ*) and loss of viable *T. solium* metacestodes (*ρ*) from the antigen and necropsy data (note that the definitions of *λ* and *ρ* vary only in the context of the different types of data). For antibody seroprevalence data, two variants of the catalytic model were fitted to data, one incorporating seroconversion only (top equation in Fig. 1a) and the other including both antibody seroconversion and seroreversion (top equation in Fig. 1b). In addition, the lack of sampling in younger ages (i.e. pigs < 6 months old) in the antibody datasets also precluded testing models including the presence/waning of maternal antibodies, which Gonzalez et al.^83^ demonstrated as persisting for up to 27 weeks after weaning. The true (unobserved) prevalence *p(a)* is a function of the catalytic models, given respectively by the equations for the simple and reversible models as

1 $$\begin{array}{c}p\left(a\right)=1- {e}^{-\lambda \left(a\right)}\end{array}$$

2 $$\begin{array}{c}p\left(a\right)=\frac{\lambda }{\lambda +\rho }\left[1- {e}^{-\left(\lambda +\rho \right)\left(a\right)}\right]\end{array}$$

The catalytic models were modified to include an age-shift model variant^84^ for models fitted to necropsy and antigen datasets. For necropsy datasets prevalence in ages < *a*_*0*_were truncated at zero given that younger animals will not have established, true infection. More specifically, the catalytic models were truncated at ages $<{a}_{0}$ at the age of 1.5 months for models fitted to necropsy data to reflect (conservatively) that cysticerci are able to mature from 60–70 days after infection^31^. For antigen datasets, catalytic models were truncated at ages $<{a}_{0}$ at the age of 0.5 months given that antigen positivity is possible from 2 weeks post-exposure^66^. The age-shift catalytic models are given by the equations for the simple and reversible models (second equation in both Fig. 1a,b) respectively as,

3 $$\begin{array}{c}p\left(a\right)=1- {e}^{-\lambda \left(a - {a}_{0}\right)}\end{array}$$

4 $$p\left(a\right)=\frac{\lambda }{\lambda +\rho }\left[1- {e}^{-\left(\lambda +\rho \right)\left(a-{a}_{0}\right)}\right]$$

Further details regarding interpretation of the parameters are provided in Fig. 1.

### Model fitting and comparison

All analyse and modelling were performed in R (https://www.r-project.org/)^85^. A likelihood was constructed assuming that the observed data (representing a binary presence/absence of markers related to exposure or infection) are a realization of an underlying binomial distribution with probability *p*(*a*) (the true (unobserved) prevalence), given by the catalytic model as previously described and adjusted to give the observed or apparent prevalence, *p’*(*a*), by the sensitivity (*se*) and specificity (*sp*) of the diagnostic used in the respective datasets. The adjustment is based on the equation^86^,

5 $$p{^{\prime}}(a)=\left(1-sp\right)+\left(se+sp-1\right)\times p\left(a\right)$$

Therefore, the likelihood of the data on the number of observed seropositive or infected pigs of age *a*, *r*(*a*), from *n*(*a*) animals tested is,

6 $$L\left( {r,n\left| \uptheta \right.} \right) = {\Pi _a}{p^\prime }{\left( a \right)^{r\left( a \right)}}{\left[ {1 - {p^\prime }\left( a \right)} \right]^{n\left( a \right){ - {r\left( a \right)}}}}$$

where θ denotes the parameters of the catalytic models and diagnostic performance (i.e. sensitivity and specificity). Where the same diagnostic was applied across surveys, the test specificity and sensitivity were jointly fitted to obtain a single posterior distribution for diagnostic sensitivity and specificity, and a setting-specific FoI (*λ* and *ρ*) posterior distribution. This approach assumes that sensitivity and specificity are uncertain but do not vary substantively by setting. We acknowledge this as a limitation of our work since, in reality, these parameters may vary among locations, partly because of other cross-reactive *Taenia* species (such as *T. hydatigena*^68–71^). However, in the absence of information on the prevalence of such species^87^—or on the relative contribution of within- and between-location variability in diagnostic performance^88,89^—we were unable to either construct location-specific priors or to estimate location-specific effects on diagnostic performance. A Bayesian Markov chain Monte Carlo (MCMC) Metropolis–Hastings sampling algorithm was implemented to obtain the parameter posterior distribution *f*(θ|*r*, *n*) , assuming a flat uniform prior for *λ*, and a flat uniform prior including limits (0,1) for *ρ*. A limit of 1 month^–1^ was used for *ρ* as this represents a minimum duration (the reciprocal of the rate) of at least 1 month that a pig can be seropositive, antigen positive or infected. Informative beta distribution priors for the diagnostic sensitivity and specificity were fitted to published estimates of the mean and 95%CIs for these parameters (noting that α and β shape parameters characterise the beta distribution, whereby α/(α + β) gives the mean of the distribution). Supplementary Table S1 and Supplementary Figure S4 show the informative beta prior distributions for diagnostic sensitivity and specificity. For jointly-fitted datasets based on the same diagnostic used between surveys, we estimated a single posterior distribution for diagnostic sensitivity and specificity, but dataset-specific FoI parameter values (*λ* and *ρ*) were estimated.

A maximum of 20,000,000 iterations were run for models fitted simultaneously to multiple (jointly-fitted) datasets, given that substantial subsampling was required to reduce autocorrelation, and a maximum of 1,000,000 iterations were run for individually-fitted datasets, with the first 10% of runs being discarded as burn-in in both cases. The parameter posterior distributions, used to generate predicted prevalence curves and associated uncertainties for each dataset, were summarised using the median and 95% Bayesian credible intervals (95% BCIs).

Model fits were compared between the simple and reversible catalytic models for individually- and jointly- fitted datasets using the deviance information criterion (DIC)^90^, with the model generating the smallest DIC score being selected.

## Supplementary information

Supplementary Information 1.

## Data Availability

Aggregated level data used in this study, obtained from the literature, can be found in a data repository through the following link: 10.14469/hpc/7447. Individual-level data additionally availability for specific studies as follows: Kungu et al.^37^: “The datasets generated during and/or analyzed during the current study are available from the corresponding author on reasonable request.” Sarti et al.^40^ : “The datasets generated during and/or analysed during the current study are available from the corresponding author on reasonable request”. Khaing et al.^49^: “The datasets generated during and/or analysed during the current study are available from the corresponding author on reasonable request.” Aggregated level data obtained through other data repositories (outside of journal publication): Fèvre et al.^49^: “The datasets generated during and/or analysed during the current study are available in the open access repository held by the University of Liverpool, [https://dx.doi.org/10.17638/datacat.liverpool.ac.uk/352].

## Code availability

Model code available from the following GitHub repository: https://github.com/Mad206/FoI_bayesian.
