# Supplementary material for: Force-of-infection of Taenia solium porcine cysticercosis: a modelling analysis to assess global incidence and prevalence trends
Source: Sci Rep. 2020 Oct 19;10:17637. doi: 10.1038/s41598-020-74007-x (PMC7572398; doi:10.1038/s41598-020-74007-x)
Supplement: Supplementary file 1 — Supplementary Information 1. [file 41598_2020_74007_MOESM1_ESM.docx]

**Supplementary File**

# **Force-of-Infection of *Taenia solium* porcine cysticercosis:**

# **a modelling analysis to assess global incidence and prevalence trends**

**Matthew A. Dixon^1,2,*^, Peter Winskill^2^, Wendy E. Harrison^3^, Charles Whittaker^2^, Veronika Schmidt^4,5^, Elsa Sarti^6^, Saw Bawm^7^, Michel M. Dione^8^, Lian F. Thomas^9,10^, Martin Walker^11^_,_**

**Maria-Gloria Basáñez^1,2^**

1 Department of Infectious Disease Epidemiology and London Centre for Neglected Tropical Disease Research (LCNTDR), Faculty of Medicine, School of Public Health, Imperial College London, London W2 1PG, UK

2 MRC Centre for Global Infectious Disease Analysis, Department of Infectious Disease Epidemiology, Faculty of Medicine, School of Public Health, Imperial College London, London W2 1PG, UK

3 SCI Foundation, Edinburgh House, 170 Kennington Lane, London, SE11 5DP

4 Department of Neurology, Center for Global Health, Technical University Munich (TUM), Munich, Germany

5 Centre for Global Health, Institute of Health and Society, University of Oslo, Oslo, Norway

6 Sanofi Pasteur Latin America, Av. Universidad N° 1738, Colonia Coyoacán 04000, México D.F., México

7 University of Veterinary Science, Yezin, Nay Pyi Taw 15013, Myanmar

8 International Livestock Research Institute, P.O. Box 24384, Kampala, Uganda

9 International Livestock Research Institute (ILRI), Old Naivasha Road, PO Box 30709-00100, Nairobi, Kenya.

10 Institute for Infection and Global Health, University of Liverpool, 8 West Derby Street, Liverpool L69 7BE, UK

11 Department of Pathobiology and Population Sciences and London Centre for Neglected Tropical Disease Research (LCNTDR), Royal Veterinary College, Hatfield AL9 7TA, UK

^*^ Corresponding author: [m.dixon15@imperial](mailto:m.dixon15@imperial).ac.uk

**Supplementary Figure S1. Published articles or age-infection data identfied using a PRISMA^1^ systematic search**

Records identified through database searching
(n = 1,809)

*PubMed: 487; Web of Science (all databases): 1,084; LILACS: 204; AJOL: 34*

Abstracts screened

(n = 343)

Titles screened
(n = 1,293)

Additional records identified through other sources
(n = 1)

## Identification

Abstracts excluded
(n = 125)

Wong parasite species: 1

Only in humans: 82

Pre-clinical/clinical research only: 7

Diagnostic development: 6

Non epidemiological study: 9

No primary data collected: 20

Records after duplicates removed
(n = 1,293)

Titles excluded
(n = 950)

Wong parasite species: 50

Non-endemic : 60

Only in humans: 146

Epidemiological studies in animals other than pigs: 3

Pre-clinical/clinical research only: 145

Diagnostic development: 70

Non epidemiological study: 22

No primary data collected: 434

Unrelated topic: 20

## Screening

Full-text articles excluded
(n = 202)

Only in humans: 20

Diagnostic paper: 4

Non epidemiological study: 4

Intervention study design (no baseline data): 11

Pre-clinical/clinical research only: 3

Review only: 8

Secondary analysis on previously analysed data: 7

Language exclusion: 7

Articles inaccessible or unable to obtain age-prevalence data from authors if age mentioned: 138

Full-text articles assessed for eligibility
(n = 219)

## Eligibility

Studies with age- (sero)prevalence data to be included (n = 15)

- Data directly from published studies (n = 12)
- Data obtained from authors/online repositories (n = 3; Kungu et al., 2017^2^; Sarti et al., 2000^3^; Fèvre et al., 2017^4^)

## Included

LILACS: Latin American & Caribbean Health Sciences Literature;

AJOL: African Journals Online

**Supplementary Figure S2. Geographical distribution of studies with porcine cysticercosis age-(sero) prevalence data included in the final analysis (n= 15) by diagnostic target.**


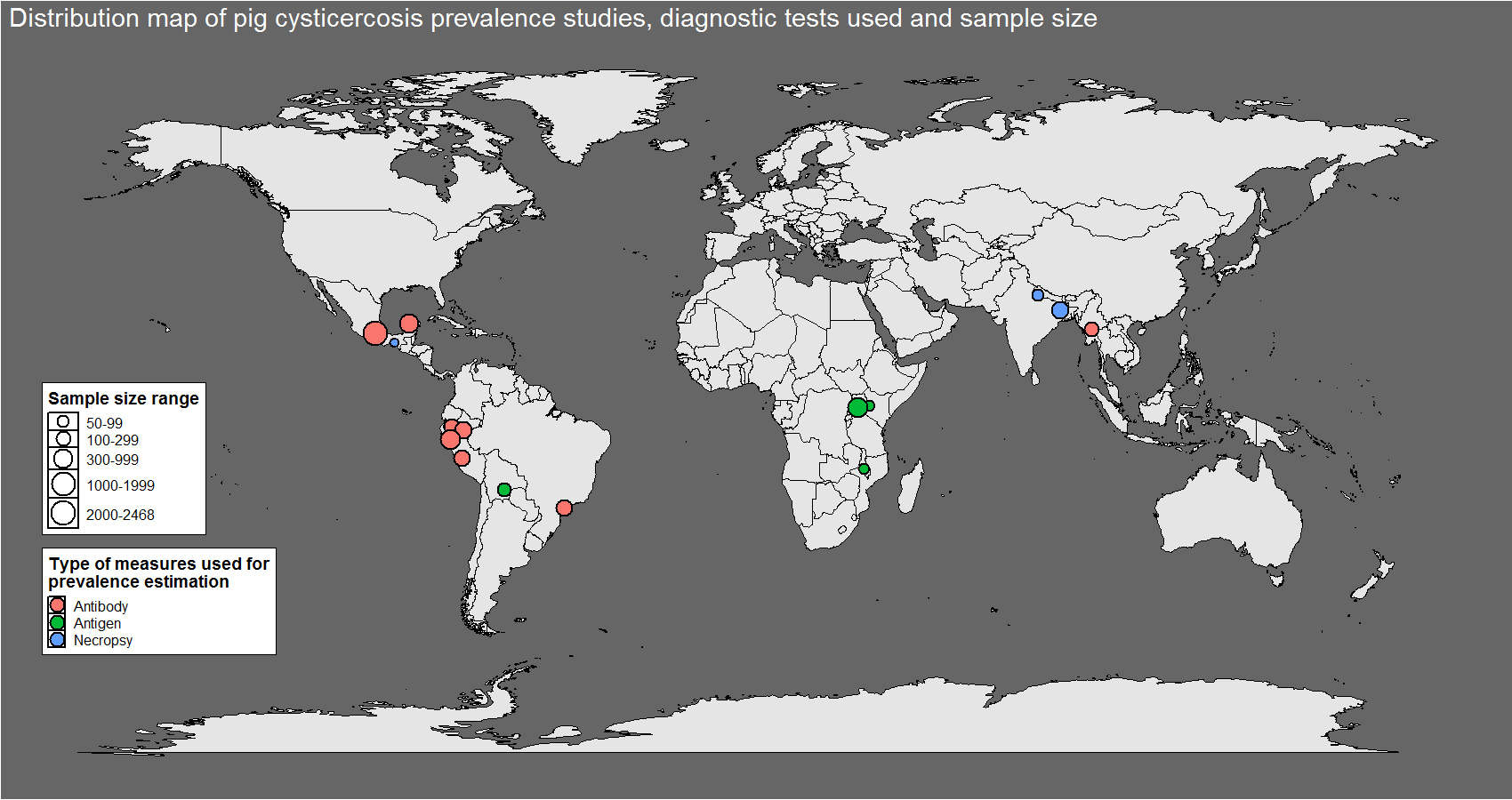
Figure produced in R programming language^5^.

**Supplementary Figure S3. Country-specific estimates of (A) the average time (in months) until pigs become antibody seropositive or infected (1/*λ*, vertical axis), and (B) the average time (in months) pigs remain antibody seropositive or infected (1/*ρ*, vertical axis)**


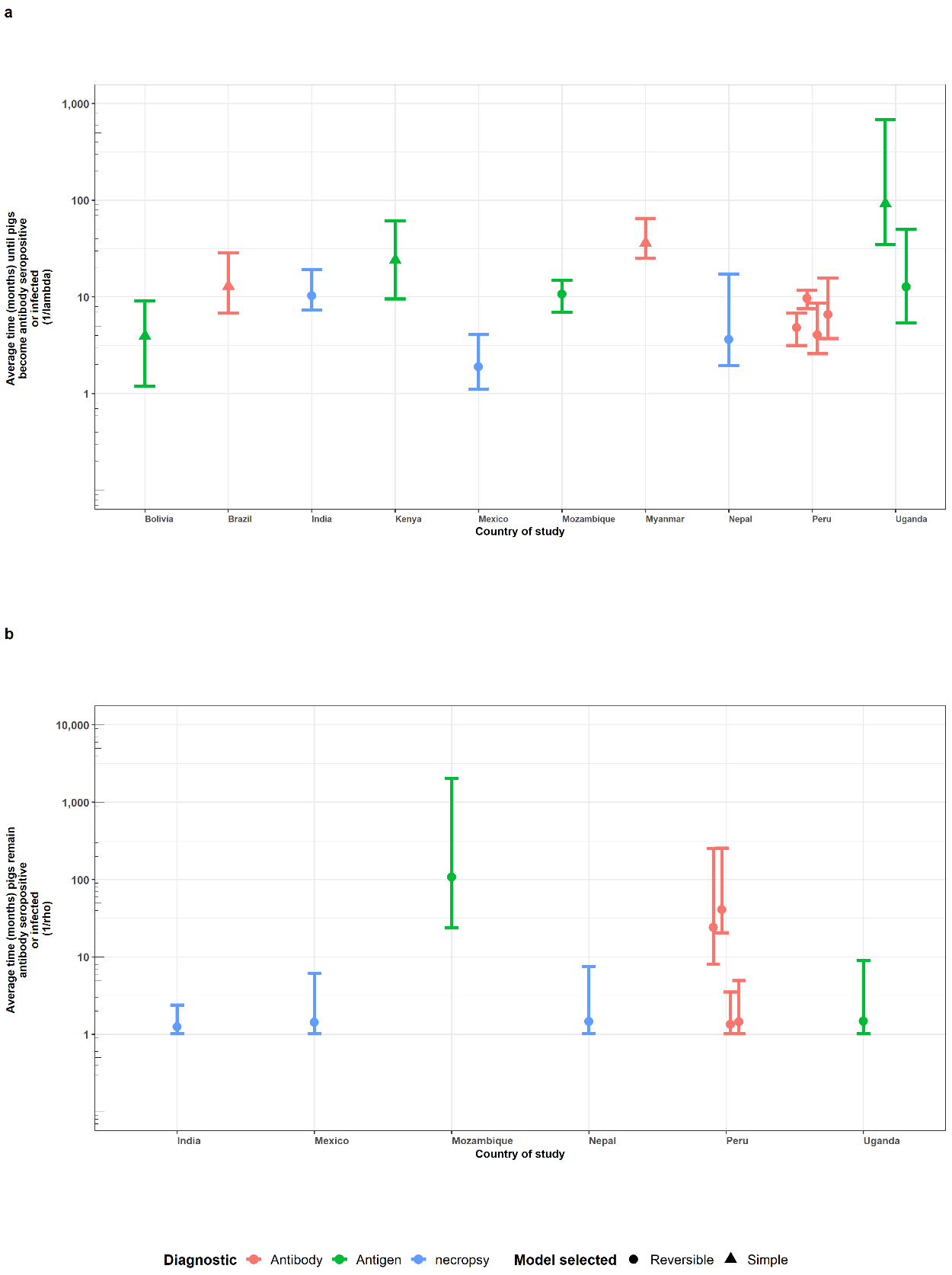


For (A) estimates are only presented where 1/λ (average duration of susceptibility in months) is less than the life expectancy of pigs. Marker colour denotes: red = antibody seroprevalence; green = antigen seroprevalence; blue = prevalence by necropsy. Solid diamonds denote the use of the reversible catalytic model; triangles are for the simple (seroconversion- or infection-only) model. Error bars are 95% Bayesian Credible Intervals around estimates.

| **Supplementary Table S1. Summary of studies included in final analysis and the diagnostic parameters used to set the probabilistic constraints for sensitivity and specificity of each test.** | | | | | | | |
| --- | --- | --- | --- | --- | --- | --- | --- |
| **Study author, year and supplementary reference** | **Location, country** | **Diagnostic** | **Sensitivity (%); specificity (%) median (95% confidence intervals given in the literature)** | **α, β shape parameters to construct each Beta distribution for sensitivity (Se) and specificity (Sp) priors (informed by column 4)** | | **Total sample size** | **Sampling strategy** |
| *Antibody detection* | | | | | | | |
| Garcia et al., 2003^6^ | Huancayo, Peru | LLGP-EITB^18,19^ | 88.8 (65.3–98.6);  48.3 (37.6–59.2)^27†^ | Se: 9.5, 1.2;  Sp: 38.6, 41.3 | | 609 | All eligible pigs in survey area |
| Jayashi et al., 2012^7^ | Piura, Peru | LLGP-EITB^18,19^ | 88.8 (65.3–98.6);  48.3 (37.6–59.2)^27†^ | Se: 9.5, 1.2;  Sp: 38.6, 41.3 | | 1,153 | All eligible pigs in survey area |
| Lescano et al., 2007^8^ | Matapalo, Peru | LLGP-EITB^18,19^ | 88.8 (65.3–98.6);  48.3 (37.6–59.2)^27†^ | Se: 9.5, 1.2;  Sp: 38.6, 41.3 | | 755 | All eligible pigs in survey area |
| Rodriguez-Canul et al., 1998^9^ | Yucatán, Mexico | Enzyme-linked immunoelectrotransfer blot (EITB) based on crude-saline extract^20^ | 93.3 (0.82–0.97);  100.0 (95.8–100.0)^9^ | Se: 27.2, 1.95;  Sp: 72.1, 0.73 | | 1,099 | Randomly selected from 3 types of husbandry system |
| Taico et al., 2003^10^ | Matapalo, Peru | LLGP-EITB^18,19^ | 88.8 (65.3–98.6);  48.3 (37.6–59.2)^27†^ | Se: 9.5, 1.2;  Sp: 38.6, 41.3 | | 440 | All eligible pigs in survey area |
| Gottschalk et al., 2006^11^ | Register microregion, São Paulo, Brazil | Enzyme-linked immunosorbent assay (Ab-ELISA) based on vesicular fluid antigen from *Taenia crassiceps*^21^ | 35.8 (26.0–41.0);  91.7 (85.0–99.0)^28^ | Se: 100, 179.3;  Sp: 77.6, 7.02 | | 551 | Limited information |
| Khaing et al., 2015^12^ | Nay Pyi Taw, Myanmar | Ab-ELISA based on Novalisa® *Taenia solium* IgG (NovaTec Immundiagnostica GmbH, Dietzenbach, Germany)^22^ | 93.8 (95% CI NA);  95.0 (95% CI NA)^12^ | Se: 103.18, 6.82;  Sp: 95.0, 5.0 | | 364 | Random sampling of households in survey area |
| Sarti et al., 2000^3*^ | Morelos, Mexico | LLGP-EITB^18,19^ | 88.8 (65.3–98.6);  48.3 (37.6–59.2)^27†^ | Se: 9.5, 1.2;  Sp: 38.6, 41.3 | | 2,468 | All eligible pigs in survey area |
| *Antigen detection* | | | | | | | |
| Carrique-Mas et al., 2001^13^ | Chuquisaca, Bolivia | HP10 Ag-ELISA; antigen ELISA using monoclonal antibodies vs excretory-secretory glycoproteins of *Taenia saginata*^23^ | 70.4 (52.7-84.7);  66.1 (44.6-85.1)^29^ | Se: 20, 8.4;  Sp: 13.2, 6.8 | | 273 | All eligible pigs in survey area |
| Pondja et al., 2015^14^ | Angónia, Mozambique | B158/B60 Ag-ELISA; antigen ELISA using monoclonal antibodies vs excretory-secretory glycoproteins of *T. saginata* ^24,25^ | 63.3 (46.8-81.6);  87.0 (78.2–94.9)^29^ | Se: 21.7, 12.6;  Sp: 60.2, 8.995 | | 282 | Piglets (aged 4 months) randomly selected and sampled at 4, 9 and 12 months of age |
| Fèvre et al., 2017^4**^ | Busia, Kenya | HP10 Ag-ELISA^23^ | 70.4 (52.7-84.7);  66.1 (44.6-85.1)^29^ | Se: 20, 8.4;  Sp: 13.2, 6.8 | | 93 | Eligible pigs from randomly selected households |
| Kungu et al., 2017^2*^ | 3 districts, Uganda | HP10 Ag-ELISA^23^ & commercial B158/B60 Ag-ELISA (apDia, Turnhout, Belgium)^26^ | *HP10 Ag ELISA*:  70.4 (52.7-84.7);  66.1 (44.6-85.1)^29^  *B158/B60 Ag-ELISA*:  63.3 (46.8-81.6);  87.0 (78.2–94.9)^30^ | *HP10 Ag-ELISA*  Se: 20, 8.4;  Sp: 13.2, 6.8  *apDia*:  Se: 21.7, 12.6;  Sp: 60.2, 8.995 | | 1,121 | 1 random pig from each randomly selected household included.  Stratified by urban (n= 245) and rural (n= 876) production systems ^††^ |
| *Necropsy* | | | | | | | |
| de Aluja et al., 1998^15^ | Mexico | Viable cysts (all cyst numbers also available) | No adjustment^ⱡ^ | |  | 52 | Limited information- non-slaughter age pigs included (< 7 months of age) |
| Sah et al., 2017^16^ | Banke, Nepal | Viable cysts | No adjustment^ⱡ^ | |  | 109 | One slaughter-age pigs sampled from each randomly selected household |
| Sasmal et al., 2008^17^ | West Bengal, India | Viable cysts | No adjustment^ⱡ^ | |  | 634 | Limited information |
| ^*^Studies for which authors provided individual-level pig infection data; ^**^age-stratified data available from the University of Liverpool open-access repository ([http://datacat.liverpool.ac.uk/352/)](http://datacat.liverpool.ac.uk/352/)%3B**); ^†^sensitivity/specificity from calculated values in Jayashi *et al*.^27^ for lentil lectin-purified glycoprotein - enzyme-linked immunoelectrotransfer blot (LLGP-EITB) reactivity to ≥ 1 band as a cut-off point for the assay; ^‡^antigen - enzyme-linked immunosorbent assays (Ag-ELISA) sensitivity and specificity calculated directly for study-specific setting (in original paper); ^††^the original analysis showed a significant difference in adjusted prevalence between the production systems, therefore data stratified on this basis. ^ⱡ^No adjustment for the necropsy method as sensitivity and specificity assumed to be 100% (see main text for a discussion on the limitations of this assumption).  NA = not available. | | | | | | | |

| **Supplementary Table S2. The deviance information criterion (DIC) and parameter estimates for simple and reversible catalytic models fitted to each observed antibody age-seroprevalence dataset (ordered by decreasing value of all-age seroprevalence). For diagnostic methods used see the corresponding study in Supplementary Table S1** | | | | | | | | | |
| --- | --- | --- | --- | --- | --- | --- | --- | --- | --- |
| **Dataset** | **All-age observed sero- prevalence (%)**  **(95% CI)** | **Catalytic model** | **DIC value** | **Diagnostic sensitivity**  **(95% BCI)** | **Diagnostic specificity**  **(95% BCI)** | ***λ* = seroconversion rate, month^-1^**  **(95% BCI)** | **1/*λ* = average time until becoming antibody seropositive (months) (95% BCI)** | ***ρ* = seroreversion rate, month^-1^ (95% BCI)** | **1/*ρ* = average time pigs remain antibody seropositive (months) (95% BCI)** |
| Jointly fitted datasets – Simple catalytic model* | | | | | | | | | |
| Garcia et al., 2003^6^ | 58.8  (54.8 – 62.7) | Simple | 100.35 | 0.735  (0.676–0.797) | 0.935  (0.922 – 0.946) | 0.253  (0.193 – 0.352) | 4.0  (2.8 – 5.2) | NA | NA |
| Jayashi et al., 2012^7^ | 45.2  (42.3 – 48.1) | Simple |  |  |  | 0.126  (0.103 – 0.152) | 8.0  (6.6 – 9.7) | NA | NA |
| Lescano et al., 2007^8^ | 26.2  (23.1 – 29.5) | Simple |  |  |  | 0.069  (0.053 – 0.087) | 14.5  (11.5 – 18.7) | NA | NA |
| Taico et al., 2003^10^ | 20.7  (17.0 – 24.8) | Simple |  |  |  | 0.047  (0.032 – 0.065) | 21.2  (15.3 – 30.9) | NA | NA |
| Sarti et al., 2000^3^ | 5.3  (4.4 – 6.2) | Simple |  |  |  | 0.0012  (0.0002 – 0.003) | > 180^‡^ | NA | NA |
| Jointly fitted datasets – Reversible catalytic model* | | | | | | | | | |
| Garcia et al., 2003^6^ | 58.8  (54.8 – 62.7) | Reversible | 84.59 | 0.889  (0.749 – 0.991) | 0.936  (0.925 –  0.946) | 0.207  (0.147 – 0.318) | 4.8  (3.1 – 6.8) | 0.042  (0.004 – 0.124) | 24.0  (8.0 – 251.1) |
| Jayashi et al., 2012^7^ | 45.2  (42.3 – 48.1) | Reversible |  |  |  | 0.104  (0.085 – 0.133) | 9.6  (7.5– 11.8) | 0.024  (0.004 – 0.049) | 41.1  (20.6 – 255.8) |
| Lescano et al., 2007^8^ | 26.2  (23.1 – 29.5) | Reversible |  |  |  | 0.247  (0.116 – 0.387) | 4.1  (2.6 – 8.6) | 0.746  (0.280 – 0.986) | 1.3  (1.0 – 3.5) |
| Taico et al., 2003^10^ | 20.7  (17.0 – 24.8) | Reversible |  |  |  | 0.152  (0.063 – 0.269) | 6.6  (3.7 – 15.8) | 0.692  (0.209 – 0.984) | 1.4  (1.0 – 4.9) |
| Sarti et al., 2000^3^ | 5.3  (4.4 – 6.2) | Reversible |  |  |  | 0.001  (0.00006 – 0.007) | > 180^‡^ | 0.63  (0.022 – 0.980) | 1.6  (1.0 – 45.8) |
| Individually-fitted datasets | | | | | | | | | |
| Rodriguez-Canul et al., 1998^9^ | 23.02  (20.6 – 25.6) | Simple | 17.51 | 0.940  (0.806–0.990) | 0.790  (0.765 – 0.82) | 0.001  (0.0001 – 0.006) | >180^‡^ | NA | NA |
| Rodriguez-Canul et al., 1998^9^ | 23.02  (20.6 – 25.6) | Reversible | 47.77 | 0.934  (0.803–0.987) | 0.991  (0.953 – 0.999) | 0.273  (0.180 – 0.367) | 3.7  (2.7 – 5.6) | 0.857  (0.562 – 0.992) | 1.2  (1.0 – 1.8) |
| Gottschalk et al., 2006^11^ | 20.5  (17.2 – 24.1) | Simple | 28.63 | 0.349  (0.297–0.403) | 0.921  (0.868 – 0.963) | 0.078  (0.035 – 0.146) | 12.9  (6.8 – 28.4) | NA | NA |
| Gottschalk et al., 2006^11^ | 20.5  (17.2 – 24.1) | Reversible | 32.68 | 0.360  (0.310–0.417) | 0.927  (0.873 – 0.967) | 0.103  (0.046 – 0.358) | 9.7  (2.8 – 22.0) | 0.034  (0.002 – 0.414) | 29.6  (2.4 – 422.8) |
| Khaing et al., 2015^12^ | 15.9  (12.3 – 20.1) | Simple | 33.94 | 0.940  (0.888–0.973) | 0.958  (0.915 – 0.985) | 0.028  (0.015 – 0.040) | 36.2  (25.0 – 65.1) | NA | NA |
| Khaing et al., 2015^12^ | 15.9  (12.3 – 20.1) | Reversible | 36.20 | 0.939  (0.885–0.975) | 0.54  (0.906 – 0.984) | 0.066  (0.024 – 0.173) | 15.2  (5.8 – 41.8) | 0.408  (0.027 – 0.964) | 2.5  (1.0 – 37.1) |
| Seroprevalence results are accompanied by 95% confidence intervals (95% CI) calculated by the Clopper-Pearson exact method. Parameter median posterior estimates are presented with 95% Bayesian credible intervals (95% BCI) and Deviance information criterion (DIC) model fitting scores;  *Diagnostic sensitivity and specificity jointly fitted for the antibody lentil lectin-purified glycoprotein enzyme-linked immunoelectrotransfer blot (Ab LLGP-EITB) assay^18,19^. ^‡^Duration more than upper limit of pig host life expectancy (15 years x 12 months = 180 month^30^), and therefore not shown.  NA = Not applicable. | | | | | | | | | |

| **Supplementary Table S3. The deviance information criterion (DIC) and parameter estimates for simple and reversible catalytic models fitted to each observed antigen age-seroprevalence dataset (ordered by decreasing value of all-age seroprevalence). For diagnostic methods used see the corresponding study in Supplementary Table S1** | | | | | | | | | |
| --- | --- | --- | --- | --- | --- | --- | --- | --- | --- |
| **Dataset** | **All-age observed sero- prevalence (%)**  **(95% CI)** | **Catalytic model** | **DIC** | **Diagnostic sensitivity**  **(95% BCI)** | **Diagnostic specificity**  **(95% BCI)** | ***λ* = rate of infection acquisition, month^-1^  (95% BCI)** | **1/*λ* = average time until pigs become infected (months)**  **(95% BCI)** | ***ρ* = rate of infection loss,**  **month^-1^**  **(95% BCI)** | **1/*ρ* = average duration of infection (months)**  **(95% BCI)** |
| Jointly-fitted datasets – Simple catalytic model* | | | | | | | | | |
| Carrique-Mas et al., 2001^13^ | 37.4  (31.6 – 43.4) | Simple | 82.43 | 0.488  (0.376–0.650) | 0.927  (0.907–0.949) | 0.254  (0.109 – 0.836) | 3.9  (1.2 – 9.1) | NA | NA |
| Fèvre et al., 2017^4^ | 18.8  (11.2 – 28.8) | Simple |  |  |  | 0.042  (0.016 – 0.105) | 24.0  (9.5 – 61.5) | NA | NA |
| Kungu et al., 2017 (urban)^2^ | HP10: 9.8  (6.4 – 14.2) | Simple |  |  |  | 0.011  (0.0015 – 0.029) | 91.8  (34.8 – 683.7) | NA | NA |
| Kungu et al., 2017 (rural)^2^ | HP10: 8.11  (6.4 – 10.1) | Simple |  |  |  | 0.003  (0.0004 – 0.011) | >180^‡^ | NA | NA |
| Jointly fitted datasets – Reversible catalytic model* | | | | | | | | | |
| Carrique-Mas et al., 2001^13^ | 37.4  (31.6 – 43.4) | Reversible | 89.57 | 0.646  (0.467–0.808) | 0.929  (0.906–0.959) | 0.539  (0.155 – 0.961) | 1.9  (1.0 – 6.5) | 0.421  (0.046 – 0.942) | 2.4  (1.1 – 24.8) |
| Fèvre et al., 2017^4^ | 18.8  (11.2 – 28.8) | Reversible |  |  |  | 0.178  (0.031 – 0.589) | 5.6  (1.7 – 32.5) | 0.666  (0.052 – 0.977) | 1.5  (1.0 – 19.3) |
| Kungu et al., 2017 (urban)^2^ | HP10: 9.8  (6.4 – 14.2) | Reversible |  |  |  | 0.038  (0.004 – 0.130) | 26.1  (7.7 – 227.6) | 0.631  (0.076 – 0.976) | 1.6  (1.0 – 13.2) |
| Kungu et al., 2017 (rural)^2^ | HP10: 8.11  (6.4 – 10.1) | Reversible |  |  |  | 0.017  (0.0007 -0.071) | 60.0  (14.2–1,379.3) | 0.699  (0.102 – 0.982) | 1.4  (1.0 – 9.8) |
| Jointly fitted datasets – Simple catalytic model** | | | | | | | | | |
| Pondja et al., 2015^14^ | 32.6  (27.2 – 38.4) | Simple | 108.64 | 0.679  (0.552–0.806) | 0.967  (0.954–0.978) | 0.089  (0.063 – 0.132) | 11.2  (7.6 – 15.8) | NA | NA |
| Kungu et al., 2017 (urban)^2^ | apDia:  9.8  (6.4 – 14.2) | Simple |  |  |  | 0.012  (0.005 – 0.022) | 83.9  (44.6 – 200) | NA | NA |
| Kungu et al., 2017 (rural)^2^ | apDia:  2.85  (1.9 – 4.2) | Simple |  |  |  | 0.0005  (0.0001– 0.002) | >180^‡^ | NA | NA |
| Jointly fitted datasets – Reversible catalytic model** | | | | | | | | | |
| Pondja et al., 2015^14^ | 32.6  (27.2 – 38.4) | Reversible | 105.20 | 0.685  (0.552–0.815) | 0.970  (0.956–0.981) | 0.093  (0.067 – 0.143) | 10.7  (7.0 -14.9) | 0.009  (0.0005–0.042) | 107.2  (23.7 – 2,034.1) |
| Kungu et al., 2017 (urban)^2^ | apDia:  9.8  (6.4 – 14.2) | Reversible |  |  |  | 0.079  (0.020 – 0.186) | 12.7  (5.4 -50.0) | 0.677  (0.112 – 0.984) | 1.5  (1.0 – 8.9) |
| Kungu et al., 2017 (rural)^2^ | apDia:  2.85  (1.9 – 4.2) | Reversible |  |  |  | 0.005  (0.0003–0.024) | >180^‡^ | 0.733  (0.122 – 0.988) | 1.4  (1.0 – 8.2) |
| Seroprevalence results are accompanied by 95% confidence intervals (95% CI) calculated by the Clopper-Pearson exact method. Parameter median posterior estimates are presented with 95% Bayesian credible intervals (95% BCI) and Deviance information criterion (DIC) model fitting scores;  *Diagnostic sensitivity and specificity for the HP10 Ag-ELISA test^23^ jointly fitted across datasets. **Diagnostic sensitivity and specificity for the B158/B60 Ag-ELISA^25^ or commercial B158/B60 Ag-ELISA (apDia, Turnhout, Belgium)^26^ jointly fitted across datasets. ^‡^Duration more than upper limit of pig host life expectancy (15 years x 12 months = 180 month)^30^, and therefore not shown.  NA = Not applicable. | | | | | | | | | |

| **Supplementary Table S4. The deviance information criterion (DIC) and parameter estimates for simple and reversible catalytic models fitted to each age-prevalence (necropsy) dataset (ordered by decreasing value of all-age prevalence)** | | | | | | | |
| --- | --- | --- | --- | --- | --- | --- | --- |
| **Dataset** | **All-age observed prevalence (%)**  **(95% CI)** | **Catalytic model** | **DIC value** | ***λ* = rate of infection acquisition, month^-1^**  **(95% BCI)** | **1/*λ* = average time until pigs become infected (months)**  **(95% BCI)** | ***ρ* = rate of infection loss,**  **month^-1^**  **(95% BCI)** | **1/*ρ* = average duration of infection (months)**  **(95% BCI)** |
| de Aluja et al., 1998^15^ | 32.7  (20.3 – 47.1) | Simple | 17.63 | 0.209  (0.127 – 0.322) | 4.8  (3.1 – 7.9) | NA | NA |
| de Aluja et al., 1998^15^ | 32.7  (20.3 – 47.1) | Reversible | 14.11 | 0.529  (0.245 – 0.896) | 1.9  (1.1 – 4.1) | 0.700  (0.163 – 0.986) | 1.4  (1.0 – 6.1) |
| Sah et al., 2017^16^ | 28.4  (20.2 – 37.9) | Simple | 19.81 | 0.027  (0.019 – 0.038) | 36.4  (26.0 – 52.3) | NA | NA |
| Sah et al., 2017^16^ | 28.4  (20.2 – 37.9) | Reversible | 19.65 | 0.276  (0.058 – 0.515) | 3.6  (1.9 – 17.4) | 0.684  (0.133 – 0.980) | 1.5  (1.0 – 7.5) |
| Sasmal et al., 2008^17^ | 10.3  (8.0 – 12.9) | Simple | 53.86 | 0.011  (0.008 – 0013) | 94.3  (75.0 – 122.8) | NA | NA |
| Sasmal et al., 2008^17^ | 10.3  (8.0 – 12.9) | Reversible | 21.57 | 0.097  (0.052 – 0.137) | 10.3  (7.3 – 19.1) | 0.801  (0.481 – 0.986) | 1.2  (1.0- 2.4) |
| Seroprevalence results are accompanied by 95% confidence intervals (95% CI) calculated by the Clopper-Pearson exact method. Parameter median posterior estimates are presented with 95% Bayesian credible intervals (95% BCI) and Deviance information criterion (DIC) model fitting scores.  NA = Not applicable.  For necropsy data it was assumed that both sensitivity and specificity were equal to 100% (see main text for a discussion of the limitations of this assumption). | | | | | | | |

**Supplementary Figure S4. Informative beta distribution priors constructed for sensitivity and specificity parameters for each diagnostic.**


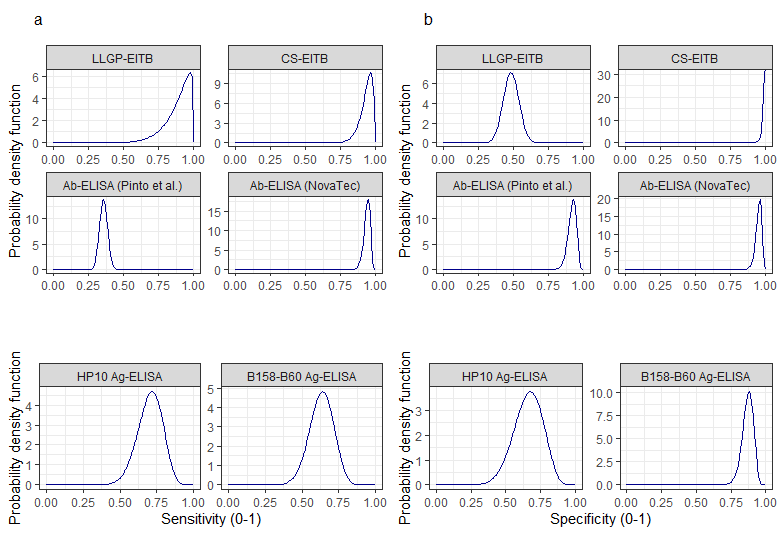


β prior distributions for: A) sensitivity (se) (all two left-hand column plots); B) specificity (sp) (all two right-hand column plots) of each diagnostic, constructed with α and β shape parameters provided in Supplementary Table S1. The β distribution provides a more flexible alternative to the uniform distribution where the parameters of interest are constrained between 0 and 1^30^. The shape parameters were fitted to the literature estimates of se and sp (whereby α/(α+β)) equals the mean of the distribution^31^).

**Supplementary References**

1. Moher, D., Liberati, A., Tetzlaff, J., Altman, D. & The PRISMA Group. Preferred reporting items for systematic reviews and meta-analyses: the PRISMA statement. *PLoS Med.* **6,** e1000097 (2009).
2. Kungu, J. M. *et al*. Sero-prevalence of *Taenia* spp. cysticercosis in rural and urban small-holder pig production settings in Uganda. *Acta Trop*. **165,** 110–115 (2017).
3. Sarti, E. *et al*. Mass treatment against human taeniasis for the control of cysticercosis: a population-based intervention study. *Trans. R. Soc. Trop. Med. Hyg*. **94,** 85–89 (2000).
4. Fèvre, E. M. *et al*. An integrated study of human and animal infectious disease in the Lake Victoria crescent small-holder crop-livestock production system, Kenya. *BMC Infect*. *Dis.* **17,** 457 (2017).
5. R Core Team. R: A language and environment for statistical computing (Version 3.6.1). *R Foundation for Statistical Computing, Vienna, Austria.* <https://www.R-project.org/> (2019).
6. Garcia, H. H. *et al*. Hyperendemic human and porcine *Taenia solium* infection in Peru. *Am. J. Trop. Med. Hyg*. **68,** 268–275 (2003).
7. Jayashi, C. M. *et al*. Seroprevalence and Risk Factors for *Taenia solium* Cysticercosis in Rural Pigs of Northern Peru. *PLoS Negl. Trop. Dis*. **6, e1733** (2012).
8. Lescano, A. G. *et al*. Swine cysticercosis hotspots surrounding *Taenia solium* tapeworm carriers. *Am. J. Trop. Med. Hyg*. **76,** 376–383 (2007).
9. Rodriguez-Canul, R. *et al*. Application of an immunoassay to determine risk factors associated with porcine cysticercosis in rural areas of Yucatan, Mexico. *Vet. Parasitol*. **79,** 165–180, [10.1016/S0304-4017(98)00161-7](https://doi.org/10.1016/S0304-4017(98)00161-7) (1998).
10. Taico U., F., López U., T., González Z., A., García L., H. & Gilman, R. Epidemiología de la cisticercosis porcina en tres caseríos de la provincia de Zarumilla, Tumbes. *Rev. Investig. Vet. Perú.* **14,** 166–173 (2003).
11. Gottschalk, S. *et al*. Seroprevalence and epidemiological aspects of cysticercosis of swine "raised in the backyard" in the Registro microregion, Sao Paulo State. *Vet. Zootec*. **13,** 192–200 (2006).
12. Khaing, T. A., Bawm, S., Wai, S. S., Htut, Y. & Htun, L. L. Epidemiological survey on porcine cysticercosis in Nay Pyi Taw area, Myanmar. *J. Vet. Med*. **2015,** 340828 (2015).
13. Carrique-Mas, J. *et al*. An epidemiological study of *Taenia solium* cysticercosis in a rural population in the Bolivian Chaco. *Acta Trop*. **80,** 229–235 (2001).
14. Pondja, A. *et al.* Prevalence and risk factors of porcine cysticercosis in Angónia District, Mozambique. *PLoS Negl. Trop. Dis*. **4,** e594 (2010).
15. de Aluja, A. S., Martinez, M. J. & Villalobos, A. N. *Taenia solium* cysticercosis in young pigs: age at first infection and histological characteristics. *Vet. Parasitol*. **76,** 71–79 (1998).
16. Sah, K. *et al*. A hyperendemic focus of *Taenia solium* transmission in the Banke District of Nepal. *Acta Trop*. **176,** 78–82 (2017).
17. Sasmal, N. K., Sarkar, A. & Laha, R. Transmission dynamics of pig cysticercosis and taeniasis in highly endemic tribal communities. *Environ. Ecol*. **26,** 76–80 (2008).
18. Tsang, V. C., Brand, J. A. & Boyer, A. E. An enzyme-linked immunoelectrotransfer blot assay and gly-coprotein antigens for diagnosing human cysticercosis (*Taenia solium*). *J. Infect. Dis*. **159,** 50–59 (1989).
19. Tsang, V. C., Gilman, R. & Pilcher, J. B. Diagnostic assay performance of the immunoblot and its impact on the epidemiology of cysticercosis in Peru. *Am. J. Trop. Med. Hyg*. **45,** 159–160 (1991).
20. Gottstein, B., Tsang, V. C. & Schantz, P. M. Demonstration of species-specific and cross-reactive components of *Taenia solium* metacestode antigens. *Am. J. Trop. Med. Hyg*. **35,** 308–313 (1986).
21. Pinto, P. S., Vaz, A. J., Germano, P. M. & Nakamura, P. M. ELISA test for the diagnosis of cysticercosis in pigs using antigens of *Taenia solium* and *Taenia crassiceps* cysticerci. *Rev. Inst. Med. Trop. São Paulo*. **42,** 67–70 (2000).
22. NovaTec. VetLine Taenia. Available at : <http://www.meditecno.pt/Upload/Product/Archive/TAEVT0420%20engl,dt,es-08092016-CS.PDF> (2016).
23. Harrison, L. J., Joshua, G. W., Wright, S. H. & Parkhouse, R. M. Specific detection of circulating surface/secreted glycoproteins of viable cysticerci in *Taenia saginata* cysticercosis. *Parasite Immunol.* **11,** 351–370 (1989).
24. Brandt, J. R. *et al*. A monoclonal antibody-based ELISA for the detection of circulating excretory-secretory antigens in *Taenia saginata* cysticercosis. *Int. J. Parasitol*. **22,** 471–477 (1992).
25. Dorny, P. *et al*. Sero-epidemiological study of *Taenia saginata* cysticercosis in Belgian cattle. *Vet Parasitol*. **88,** 43–49 (2000).
26. Cysticercosis AG ELISA. Available at [http://apDiagroup.com/index.php?option=com_content&view=article&id=121&Itemid=313](http://apdiagroup.com/index.php?option=com_content&view=article&id=121&Itemid=313) (2019).
27. Jayashi, C. M. *et al*. Validity of the Enzyme-linked Immunoelectrotransfer Blot (EITB) for naturally acquired porcine cysticercosis. *Vet. Parasitol*. **199,** 42–49 (2014).
28. Dorny, P. *et al*. A Bayesian approach for estimating values for prevalence and diagnostic test characteristics of porcine cysticercosis. *Int. J. Parasitol*. **34,** 569–576 (2004).
29. Krecek, R. C. *et al*. Corrigendum to Prevalence of *Taenia solium* cysticercosis in swine from a communi-ty-based study in 21 villages of the Eastern Cape Province, South Africa [*Vet. Parasitol*. **154,** 38–47 (2008)]. *Vet Parasitol*. **183,** 198–200 (2011).
30. CRCNPB Australia. Invasive Species Compendium: *Sus scrofa* [ISC] (feral pig).CAB International. Accessed: 08/10/19.
31. Lambert, B. *A Student’s Guide to Bayesian Statistics.* Chapter 8 pp. 173–174 (SAGE Publications, London, 2018).
